# Inhibition of livin overcomes radioresistance in nasopharyngeal carcinoma cells

**DOI:** 10.1371/journal.pone.0229272

**Published:** 2020-03-02

**Authors:** Fei Ma, Xia Gu, Jiang-Qi Liu, Li-Hua Mo, Gui Yang, Xiao-Rui Geng, Zhi-Qiang Liu, Zhi-Gang Liu, Ping-Chang Yang

**Affiliations:** 1 Department of Traditional Chinese Medicine, Affiliated Shenzhen Maternity & Child Healthcare Hospital, Southern Medical University, Shenzhen, China; 2 Research Center of Allergy & Immunology, Shenzhen University School of Medicine, Shenzhen, China; 3 Department of Respirology, Affiliated Hospital of Xinan Medical University, Luzhou, China; 4 Department of Allergy, Longgang ENT Hospital & Shenzhen Key Laboratory of ENT, Institute of ENT, Shenzhen, China; 5 Department of Otolaryngology, Longgang Central Hospital, Shenzhen, China; 6 Guangdong Provincial Key Laboratory of Regional Immunity and Diseases, Shenzhen, China; Duke University School of Medicine, UNITED STATES

## Abstract

**Background and aims:**

Radiotherapy is one of the major remedies for the treatment of cancer, including nasopharyngeal carcinoma (NPC). Radioresistance occurs very often in target cells that is a large drawback in cancer treated with radiotherapy. Livin involves the over-growth of cancer cells. This study aims to investigate the role of livin in the radioresistance formation in NPC cells.

**Methods:**

NPC cell lines were exposed to small doses of irradiation to establish a cell model of radioresistance, in which the role of livin in the development of radioresistance was evaluated.

**Results:**

The expression of livin was observed in NPC cells, which was significantly increased after exposing to small doses of irradiation. A negative correlation was detected between livin and Fas expression in NPC cells. Livin formed a complex with heat shock factor-1 (HSF1, the transcription factor of Fas) in NPC cells after irradiation, which sped up ubiquitination of HSF1. Livin was involved in suppressing Fas expression in NPC cells with radioresistance. Exposure to livin inhibitors prevented radioresistance development and overcame the established radioresistance in NPC cells.

**Conclusions:**

Livin expression in NPC cells plays a critical role in the development of radioresistance. Depletion of livin increases the sensitiveness of NPC cells to irradiation. Target therapy against livin may have the translational potential for the treatment of NPC.

## Introduction

Nasopharyngeal carcinoma (NPC) is one of the leading causes of human death; its pathogenesis is not clear [1]. The prevalence of NPC is 0.2–0.5/100,000 people in the USA but reaches 25-50/100,000 people in the southern areas of China [2]. Because of the specific anatomical location, it is not easy to diagnose NPC at early stages, many cases are diagnosed at the advanced stage [1, 2]; some NPC patients even have metastasis in the neck or/and remote organs at the diagnosis [3]. Radiotherapy is the first choice for NPC treatment [4]. NPC cells are relatively sensitive to radiotherapy [4]. A large portion of NPC can be clinically cured by radiotherapy [4]. However, a substantial portion of NPC cells develops radioresistance in the course of radiotherapy, which seriously affects the efficacy of radiotherapy in NPC [5]. Yet, the mechanism of radioresistance development is not fully understood.

One of the mechanisms by which radiotherapy treats cancer is to induce target cell apoptosis [6, 7]. In the case of radioresistance, cancer cells may develop the defects of apoptosis, such as administration with small doses of irradiation [8]. Apoptosis is a physiological phenomenon, by which the unwanted cells, damaged cells and senescent cells are eliminated from the body [9]. Apoptosis also can be regulated. Many factors have effects on speeding apoptosis, such as tumor necrosis factor-α [10], or inhibiting apoptosis, such as Bcl2L12 [11]. Livin is a gene that encodes one of the proteins of the group-inhibitors of apoptosis (IAP) in cancer cells to render cancer cells to overgrow [12]. Yet, whether livin is involved in radioresistance development remains un-investigated.

A variety of approaches have been tried to induce apoptosis in cancer cells [13]. Hypoxia-inducible factor-1α can promote the TNF-α induced apoptosis [14]. Nitric oxide can regulate the processes of apoptosis [15]. These factors have effects on overcoming the radioresistance of cancer by speeding cancer cell apoptosis. The efficiency of regulating radioresistance on cancer cells are not satisfactory currently [16]. Since livin plays an important role in cancer cell overgrowth and inhibition of apoptosis [17], we hypothesize that to inhibit livin may regulate radioresistance in NPC cells. Therefore, in this study, the association between NPC cell radioresistance and the expression of livin NPC cells was investigated. The effects of inhibiting livin on overcoming the radioresistance of NPC cells were evaluated.

## Materials and methods

### Reagents

RNAi kit of livin, antibodies of livin, Fas, HSF1, Pol II and ubiquitin were purchased from Santa Cruz Biotech (Santa Cruz, CA). FITC labeled anti-Fas antibody was purchased from BD Biosciences (Franklin Lakes, NJ). ChIP kit, materials for immunoprecipitation and Annexin V kit was purchased from Sigma Aldrich (St. Louis., MO). Reagents and materials for RT-qPCR and Western blotting were purchased from Invitrogen (Carlsbad, CA).

### NPC cell culture

NPC cell lines, NP69 (ATCC), CNE1 cells and SUNE1 cells (obtained from Sun Yat-sen University Cancer Center) were cultured in DMEM supplemented with 10% heat inactivated fetal calf serum, 100 U/ml penicillin, 0.1 mg/ml streptomycin and 2 mM glutamine at 37°C and 5% CO_2_. The medium was changed daily. Viability of cells was greater than 99% as assessed by Trypan blue exclusion assay.

### Treating NPC cells with radiation

Based on the notion that cancer cells can acquire radioresistance by low-dose fractionated radiation within a short period [8, 18], we developed a radioresistance model. NPC cells were irradiated with γ-rays; the plates with NPC cells were exposed to radiation from a GammaCell 40 ^137^Cs irradiator (dose rate, 0.82 Gy/min) at 1 Gy daily for consecutive 4 days, which is designated “1×4 irradi” in this paper. 24 h later, the cells were used for further experiments. Following the 1×4 irradi, NPC cells were irradiated at 20 Gy one time. The cells were analyzed 24 h later.

### NPC cell colony formation assay

NPC cells were seeded into 10 cm-plates and irradiated with 2 Gy. After the colony formed, the cells were fixed and stained with crystal violet. The images were captured randomly. The Photoshop software (version 2019) was used to count the colony numbers and calculate the colony areas.

### Flow cytometry

Cells were collected from relevant experiments and stained with fluorescence-labeled anti-Fas antibodies (1:100 in dilution) or isotype IgG. The cells were analyzed with a flow cytometer (FACSCanto II, BD Bioscience). The data were analyzed with FlowJo software package (TreeStar Inc., Asland, OR).

### Assessment of apoptosis

Cells were harvested from the culture, stained with propidium iodide (PI) and annexin v reagents following the manufacturer’s instructions. The cells were analyzed with a flow cytometer (FACSCanto II). Cells stained annexin v^+^ or PI^+^ annexin v^+^ were regarded as apoptotic.

### Preparation of protein extracts

Cells were collected from relevant experiments and lysed with a lysis buffer (10 mM HEPES; 1.5 mM MgCl2; 10 mM KCl; 0.5 mM DTT; 1 mM EDTA; 0.05% NP40). The lysates were centrifuged at 10,000 g for 10 min. The supernatant was collected to be cytosolic extracts. The pellets were resuspended in a nuclear lysis buffer (5 mM HEPES; 1.5 mM MgCl_2_SO_4_; 4.6 M NaCl; 0.2 mM EDTA; 0.5 mM DTT; 26% glycerol) and stayed for 30 min. The lysates were centrifuged at 10,000 g for 10 min. The supernatant was collected to be nuclear extracts. All the procedures were performed at 4°C.

### Western blotting

Proteins were extracted from NPC cells and fractioned by SDS-PAGE and transferred onto a PVDF membrane. The membrane was blocked by incubating with 5% skim milk for 30 min, incubated with the primary antibodies (1:500) overnight at 4°C, washed with TBST (Tris-buffered saline containing 0.1% Tween 20) 3 times, incubated with the secondary antibodies (labeled with peroxidase; diluted 1:10,000) for 2 h at room temperature, washed with TBST for 3 times. Immunoblots on the membrane were developed by the enhanced chemiluminescence and photographed in an imaging device.

### Immunoprecipitation (IP)

Proteins were precleared by incubating with protein G agarose beads for 2 h. The beads were removed by centrifugation at 5,000 g for 5 min. The supernatant was incubated with antibodies of interest or isotype IgG overnight to form immune complexes. The complexes were precipitated by incubating with protein G agarose beads for 2 h. The beads were collected by centrifugation at 5,000 g for 5 min. Proteins on the beads were eluted with an eluting buffer and analyzed by Western blotting. All the procedures were performed at 4°C.

### Detection of ubiquitinated HSF1

Proteins were processed with the procedures of the IP. The membrane was stained with anti-HSF1 Ab first. The HSF1 blots were developed and images were taken. The membrane was treated with stripping buffer to remove the anti-HSF1 Ab and followed by staining with anti-ubiquitin Ab. The ubiquitin blot images were developed and recorded.

### Real-time quantitative RT-PCR (RT-qPCR)

Total RNA was extracted from NPC tissues or cells collected from relevant experiments with TRIzol reagent. The RNA was converted to cDNA with a reverse transcription kit following the manufacturer’s instructions. The samples were amplified on a qPCR device (CFX96 Touch Real-time PCR Detection system; Bio-Rad) with the SYBR Green Master Mix and the presence of primers of Fas (ccggacccagaataccaagt and gaagacaaagccaccccaag) and livin (agaggaggaagaggaggagg and acctcaccttgtcctgatgg). The results were processed with the 2^-ΔΔCt^ method and presented as fold change against the control group.

### Chromatin IP (ChIP)

Cells collected from relevant experiments were fixed with 1% paraformaldehyde for 15 min to cross link chromatin with the surrounding proteins. The cells were lysed with a lysis buffer and followed by sonication to shear the DNA into small pieces. The samples were then processed with the procedures of the IP. After eluting from agarose beads, DNA was recovered from the samples with a DNA extracting kit following the manufacturer’s instructions. The DNA was analyzed by qPCR in the presence of primers of the Fas promoter (actggtggtaagtgcagtga and acctcactctgcaacctctc). All the procedures were performed at 4°C.

### Inhibition of livin in NPC cells with livin inhibitor

A livin inhibitor peptide was synthesized by Gene Script (Nanjing, China) based on the amino acid sequence (GSGCGCFVRGRIVRIRCVILLLRVLRS [19]). NP69 cells were cultured in the presence or absence of this peptide at 10 μg/ml combination with irradiation.

### Statistics

Each experiment was performed at least 6 times with each time in triplicate. The difference between two groups was determined by Student *t* test. ANOVA followed by the Dunnett test or Bonferroni test was performed for multiple comparisons. The correlation between data of the two groups was performed with Pearson Correlation assay. P<0.05 was set as the significant criterion.

## Results

### Radioresistance is associated with the expression of livin in NPC cells

**Fig 1:**
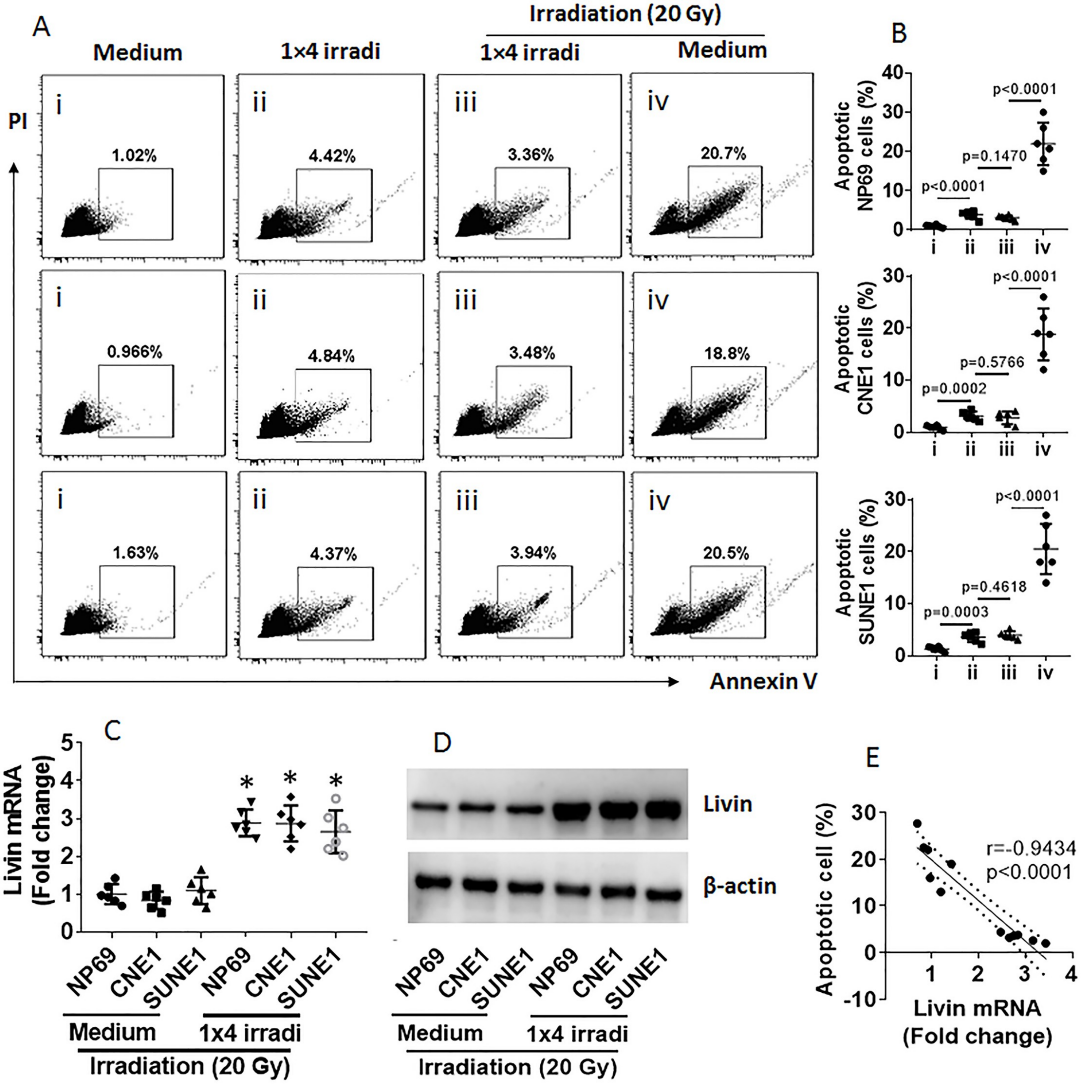
Radioresistance of NPC cells negatively correlates with expression of livin. Three NPC cell lines were irradiated at 1 Gy daily for 4 times (1×4 irradi). NPC cells with or without the 1×4 irradi were irradiated at 20 Gy and analyzed by flow cytometry, RT-qPCR and Western blotting. A, gated dot plots show apoptotic NPC cells. B, scatter dot plots show summarized apoptotic cells. C, scatter dot plots show mRNA levels of livin. D, representative immunoblots show livin protein levels. E, a negative correlation between apoptotic NPC cells and expression of livin in NPC cells. Data of bars of panel B are presented as mean ± SEM. Each dot present data obtained from an independent experiment. Statistics: ANOVA + Bonferroni test (B, C) and Pearson correlation assay (E). The data represent 6 independent experiments.

Published data indicate that livin plays an important role in cancer survival [20]. We wondered if the development of radioresistance is associated with livin expression. To test this, we developed a radioresistance model by irradiating NPC cell lines, including NP69 cells, CNE1 cells and SUNE1 cells, at 1 Gy daily for 4 times (hereafter, 1×4 irradi). As tested by irradiating NPC cells with a large dose of irradiation (20 Gy) on day 5, 1×4 irradi significantly protected NPC cells from irradiation-induced apoptosis (Fig 1A and 1B). In other words, 1×4 irradi induced radioresistance in NPC cells. We also observed that 1×4 irradi markedly induced livin expression in NPC cells (Fig 1C and 1D), which was negatively correlated with the 20 Gy irradiation-induced NPC cell apoptosis (Fig 1E). The results suggest that livin is associated with radioresistance development in NPC cells.

### Livin expression is positively correlated with NPC cell colony formation

**Fig 2:**
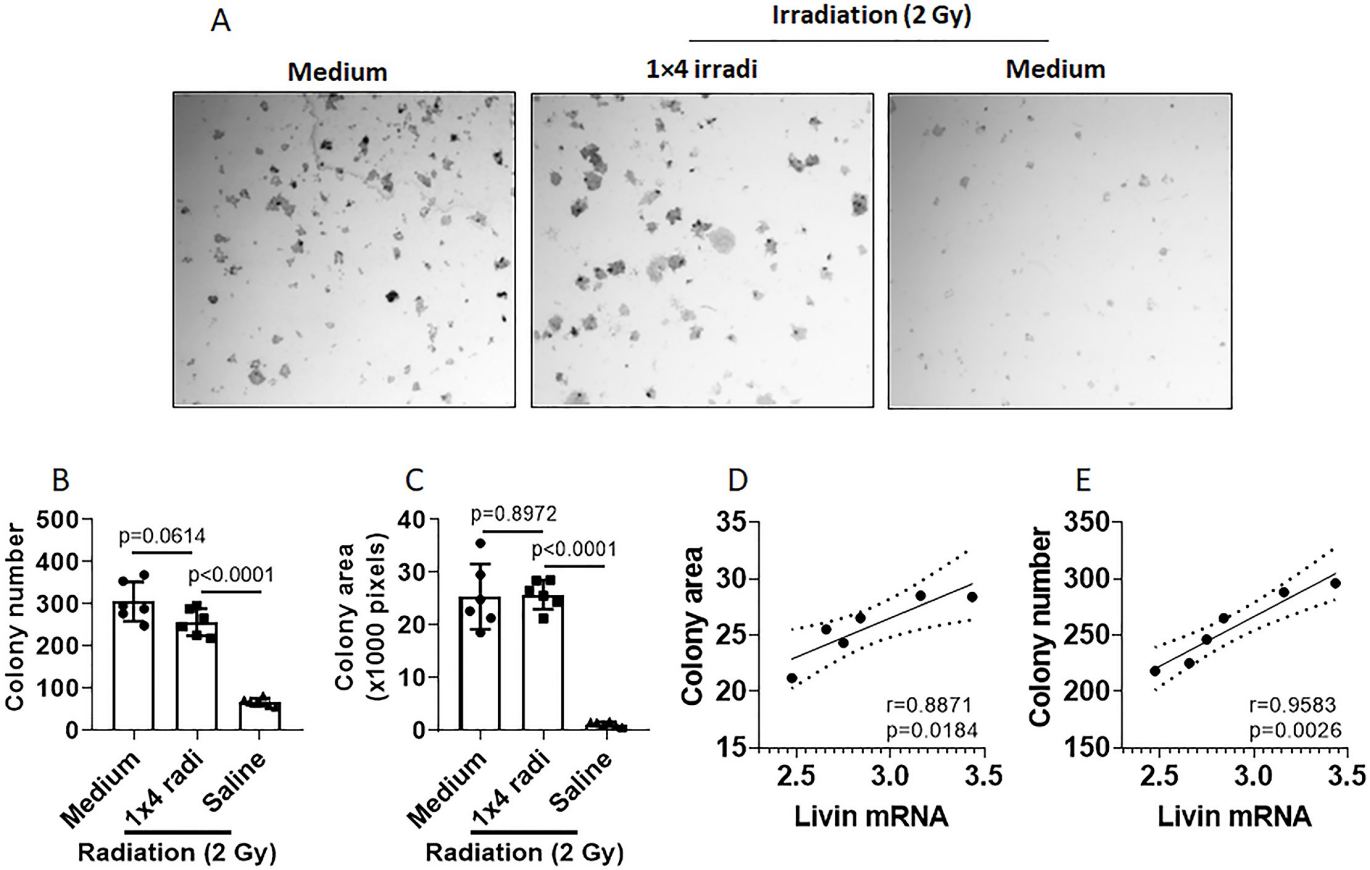
NPC cell colony formation assay. NPC cells (NP69 cells) were cultured and treated with the procedures denoted above each subpanel of A. A, representative images of the NPC colonies. B-C, 10 images were randomly taken from each group. Colony numbers were counted and colony areas were calculated with Photoshop software. B, average colony numbers per image. C, average colony areas per image. D-E, correlation assay results between livin mRNA and colony area (D) and colony number (E). The data of B and C are presented as mean ± SEM. Statistics: ANOVA + Bonferroni test (B, C) and Pearson correlation assay (D, E). The data represent 6 independent experiments.

We next tested the association between livin expression and colony formation in NPC cells. NPC cells were treated with 1×4 irradi and then exposed to 2-Gy irradiation. The cells were cultured for colony formation assay. The results showed that 1×4 irradi markedly promoted the NPC cell colony formation (Fig 2A–2C). The livin expression was positively correlated with the colony formation (Fig 2D and 2E).

### Irradiation-induced livin interferes with the expression of Fas in NPC cells

**Fig 3:**
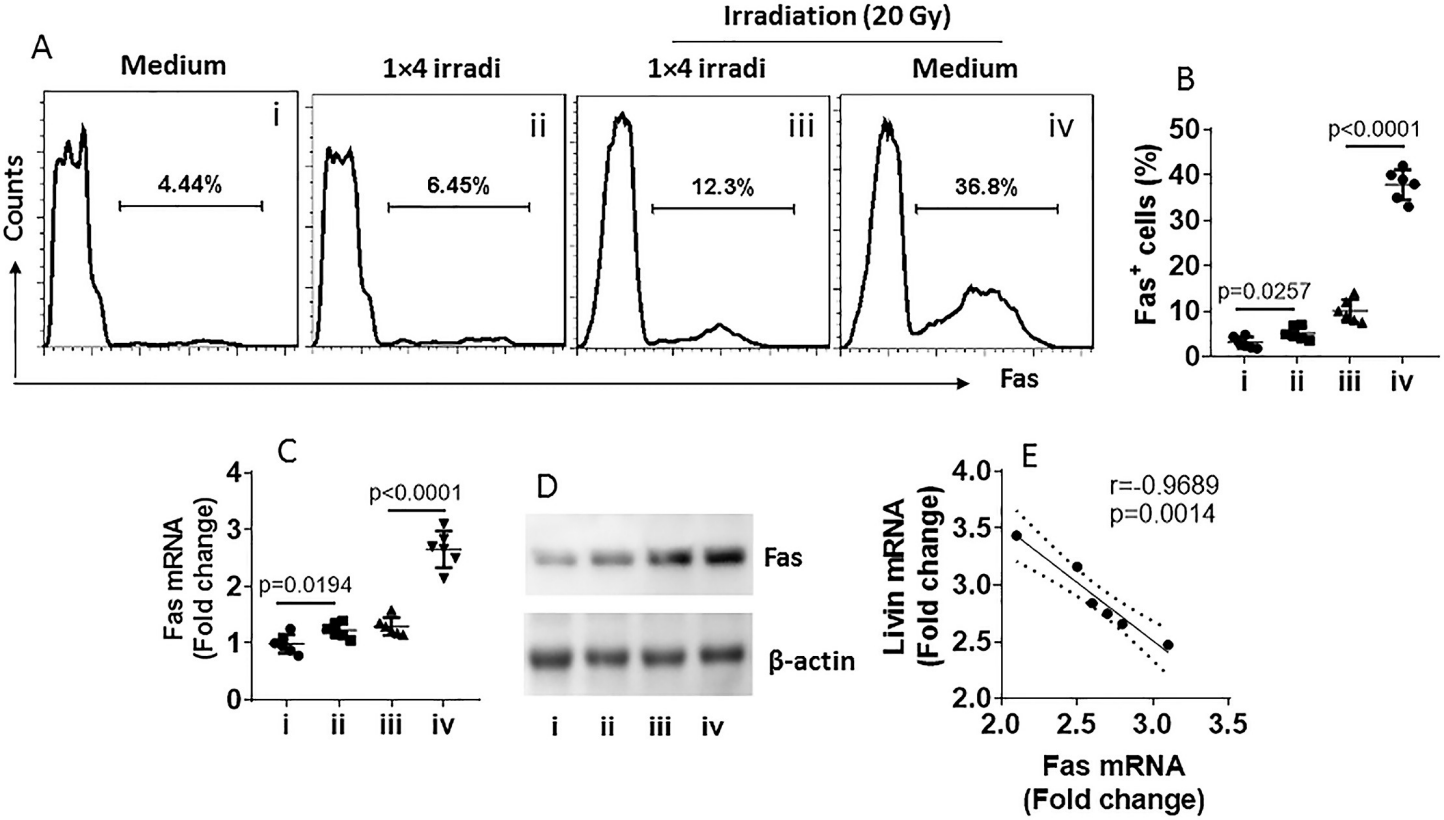
Irradiation-induced livin expression negatively correlates with Fas expression in NPC cells. A, NP69 cells were irradiated as denoted above each subpanel. Gated histograms show frequency of Fas^+^ NP69 cells. B, summarized Fas^+^ NP69 cells. C, Fas mRNA levels in the NP69 cells. D, Fas protein levels in the NP69 cells. E, a negative correlation between Fas mRNA and livin mRNA. Data of bars of B and C are presented as mean ± SEM. Each dot presents data obtained from one independent experiment. Statistics of B and C: ANOVA + Bonferroni test and Pearson correlation assay (E). The group labels of B-D are the same as panel A.

Since reduction of Fas expression in target cells is associated with the development of radioresistance [21], we then assessed the effects of livin on Fas expression in NPC cells. NPC cells were treated with 1×4 irradi. As analyzed by flow cytometry, RT-qPCR and Western blotting, Fas expression in NPC cells was reduced by irradiation in a dose-dependent manner (Fig 3A–3D). A negative correlation was detected between Fas and livin expression in irradiated NPC cells (Fig 3E). The results imply that livin may interfere with Fas expression in NPC cells.

### Livin interacts with Fas gene transcription factor in NPC cells

**Fig 4:**
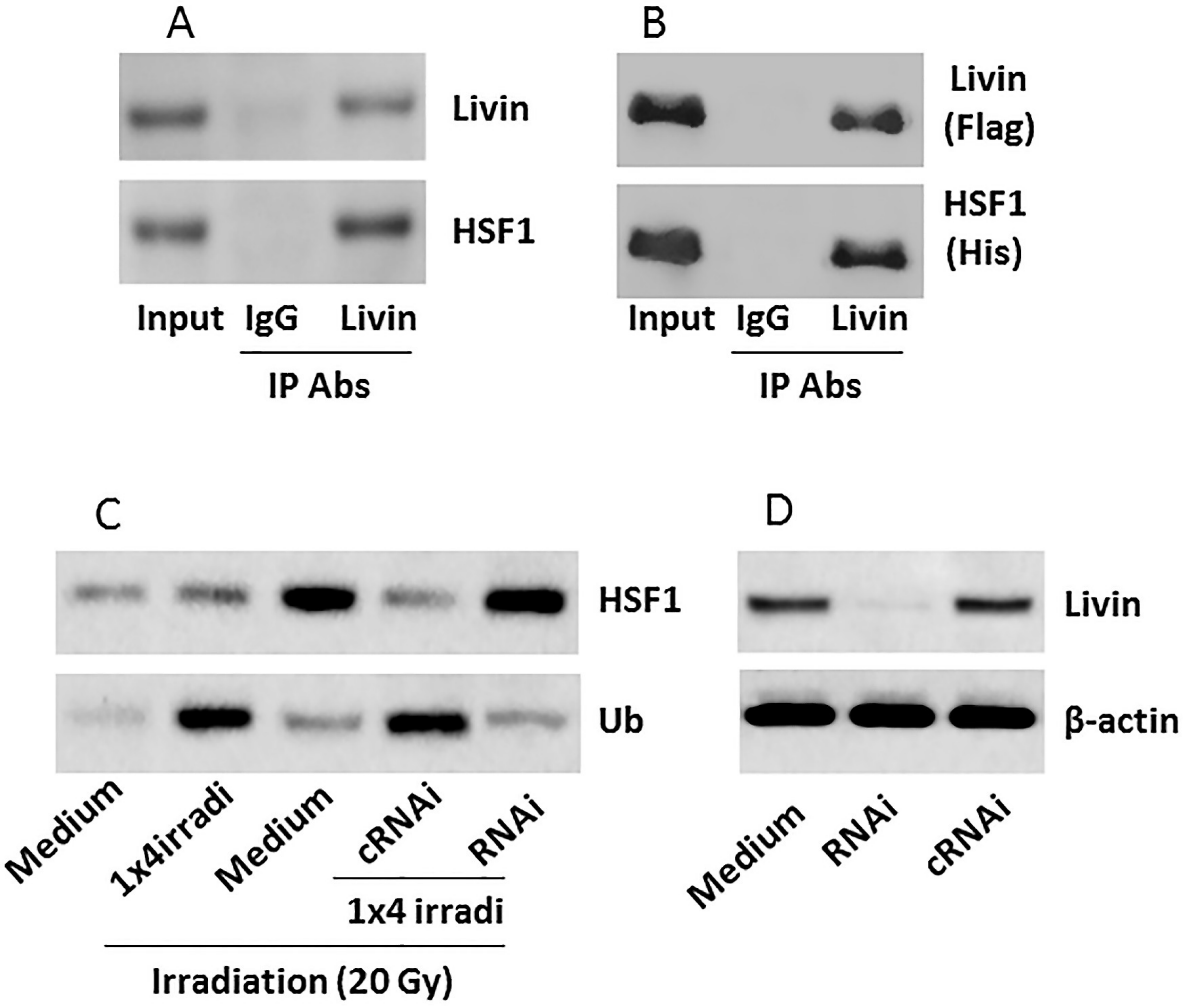
Livin forms a complex with HSF1 in NP69 cells to cause HSF1 ubiquitination. A, after treating with 1×4 irradi, NP69 cells were processed for IP (immunoprecipitation); anti-livin Ab was used as the precipitation Ab. Immunoblots show a complex of livin/HSF1. B, HEK293 cells were transfected with livin-expressing plasmids and HSF1-expressing plasmids. The HEK293 cells were processed for IP; anti-livin Ab was used as the precipitation Ab. Immunoblots show a complex of rHSF1/rlivin. C, upper immunoblots show HSF1 protein levels and lower bands show ubiquitin-positive staining in TTP protein in NP69 cells after treating with the procedures denoted below. The cells were harvested 24 h after the 20 Gy irradiation and analyzed by Western blotting. RNAi (cRNAi): Livin RNAi (control RNAi). D, results of livin RNAi in NP69 cells. The data represent 6 independent experiments.

Next, we assessed the interaction between livin and heat shock factor-1 (HSF1), the gene transcription factor of Fas [22], in NPC cells. NP69 cells were treated with 1×4 irradi. A complex of HSF1 and livin was found in NP69 cells (Fig 4A), indicating that livin can bind HSF1 to form a complex. The results were reproduced with recombinant (r) HSF1 and rlivin in HEK293 cells (Fig 4B). Treatment with 1×4 irradi also increased HSF1 ubiquitination in NP69 cells (Fig 4C), which was abolished by depletion of livin (Fig 4C and 4D). The results demonstrate that livin can physically contact the gene transcription factor, HSF1, in NP69 cells and caused HSF1 ubiquitination.

### Livin prevents Fas gene transcription in NPC cells

**Fig 5:**
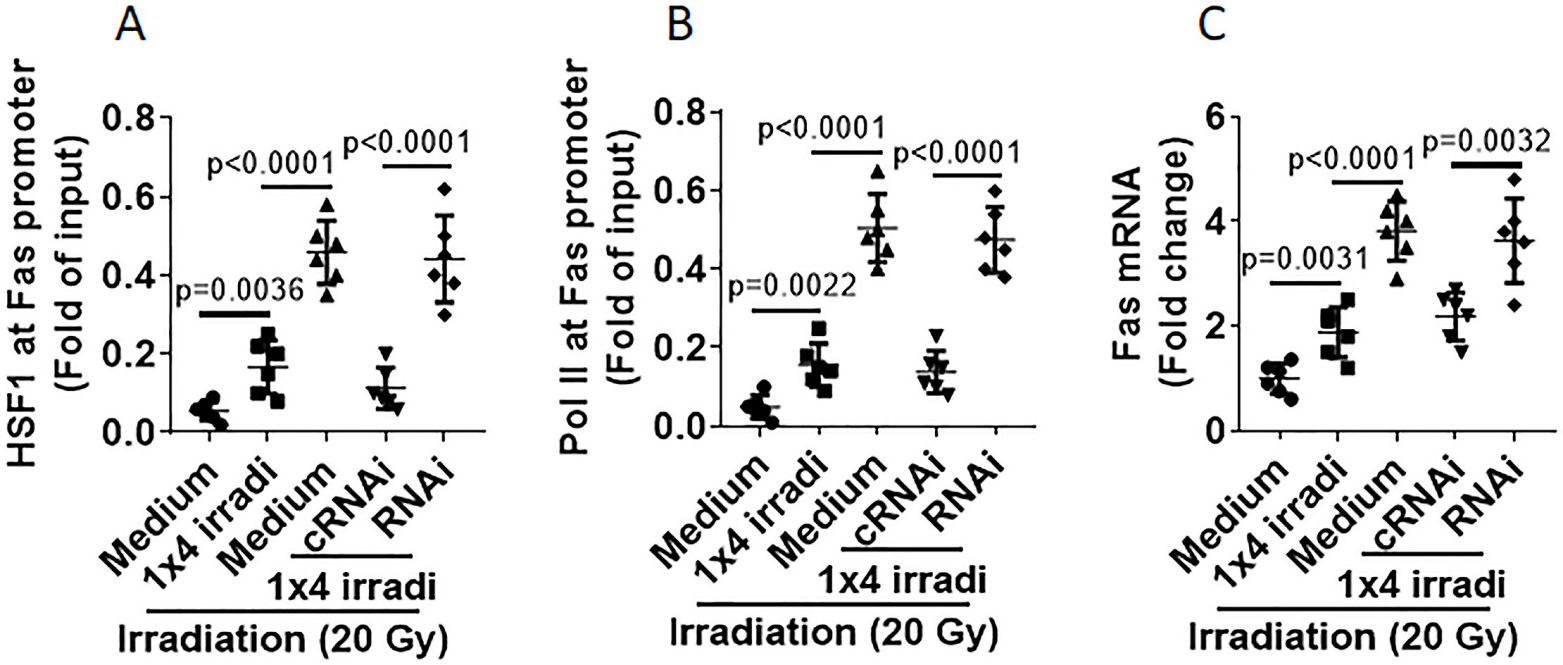
Livin interferes with irradiation-altered Fas gene transcription in NP69 cells. NP69 cells were treated with the procedures denoted on the x axis. The cells were harvested 24 h after irradiation and analyzed by ChIP and RT-qPCR. A-B, levels of HSF1 and Pol II at the Fas promoter locus. C, Fas mRNA levels. Data of bars are presented as mean ± SEM. Each dot presents data obtained from an independent experiment. Statistics: ANOVA + Bonferroni test. The data represent 6 independent experiments.

Then, we assessed the effects of livin on Fas gene transcription activities in NPC cells in response to irradiation. We observed that irradiation markedly increased HSF1 levels at the Fas promoter locus in NP69 cells, which was significantly reduced by pre-treating with 1×4 irradi; the latter could be prevented by depletion of livin (Fig 5A). The activities of RNA polymerase II (Pol II) at the HSF1 promoter locus and Fas mRNA levels were also altered by irradiation in parallel to HSF1 levels in NPC cells (Fig 5B and 5C). The results demonstrate that livin plays a crucial role in the 1×4 irradi-altered Fas gene transcription in NPC cells.

### Inhibition of livin overcomes radioresistance in NPC cells caused by irradiation at small doses

**Fig 6:**
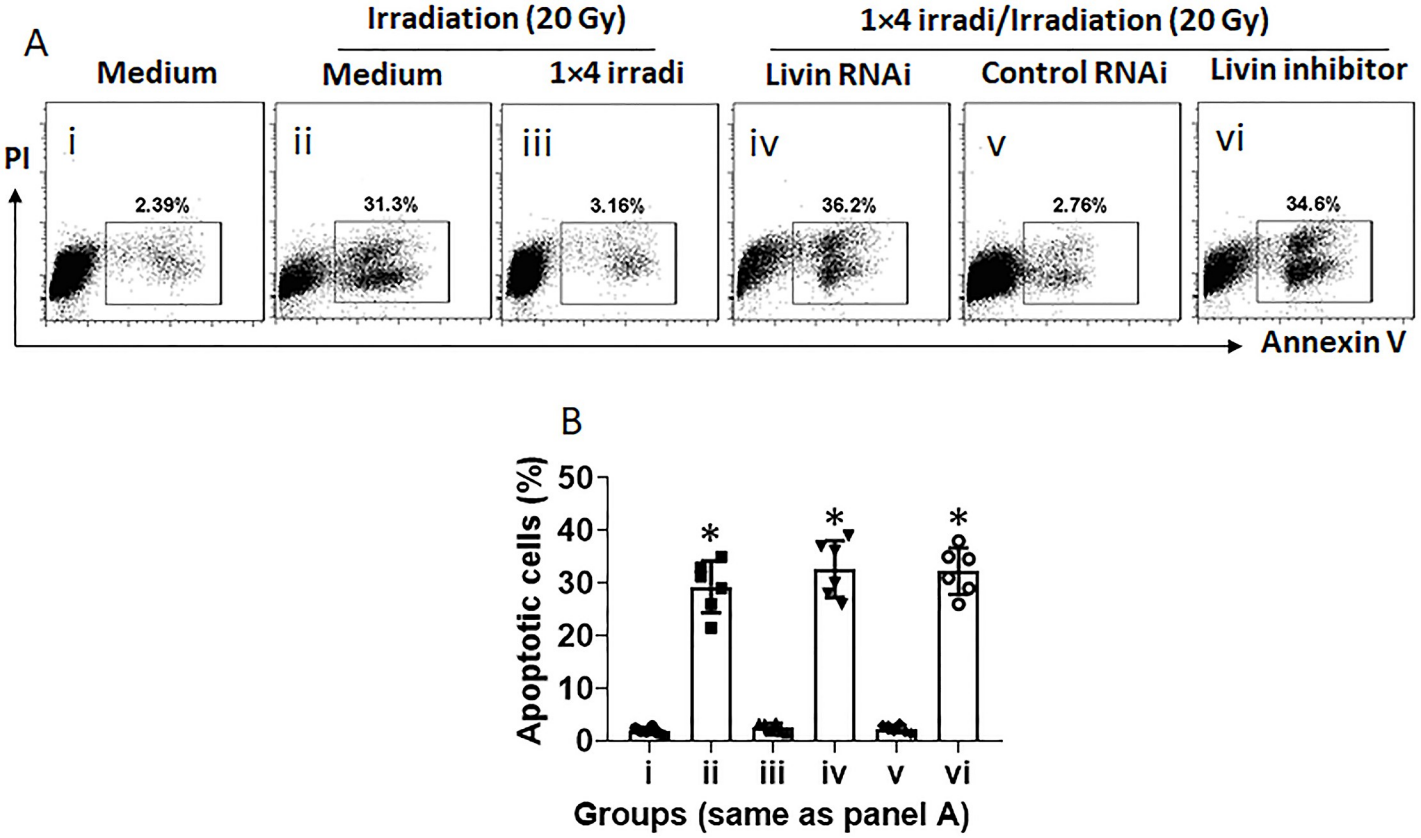
Inhibition of livin overcomes radioresistance in NPC cells. NP69 cells were treated with the 1×4 irradi to develop the radioresistance. The cells were then treated with or without livin RNAi or livin inhibitor, followed by a large dose of irradiation (20 Gy). The cells were analyzed by flow cytometry. A, gated dot plots show frequency of apoptotic NP69 cells. B, summarized data of apoptotic cells. Data of bars are presented as mean ± SEM. Each dot presents data obtained from one independent experiment. Statistics: ANOVA + Dunnett’s test. The data represent 6 independent experiments.

Data of Figs 1–5 demonstrate that livin plays a crucial role in the development of radioresistance in NPC cells. The data suggest that inhibition of livin may overcome the radioresistance. To test this, the radioresistance was developed in NP69 cells by exposing to 1×4 irradi (Fig 6A and 6B). Livin-deficient NPC cells were treated with 1×4 irradi and followed by a large dose of irradiation. The results showed that depletion of livin abolished the radioresistance in NPC cells (Fig 6C and 6D). On the other hand, the radioresistance was established in NPC cells first by 1×4 irradi. The cells were then treated with a livin inhibitor, in the culture. The cells were then treated with a large dose of irradiation (20 Gy). The results showed that treatment with livin inhibitor significantly increased the sensitiveness to radiotherapy as markedly more apoptotic cells were induced (Fig 6E and 6F). The results demonstrate that blocking livin can overcome the radioresistance in NPC cells caused by small doses of irradiation.

## Discussion

Radioresistance is a large drawback in radiotherapy for NPC and many other cancers [16]. Defects of apoptosis are one of the causes in radioresistance development [23]. Currently, there are not many effective remedies available using to adjust the defects of apoptosis and the radioresistance [23]. The present study revealed that livin played a critical role in the induction of defects of apoptosis, promotion of colony formation in NPC cells and radioresistance development. By depletion of livin expression or using livin inhibitory peptides, the irradiation-induced defects of apoptosis and the development of radioresistance were abolished in NPC cells.

Radioresistance commonly occurs in cancer cells in the course of radiotherapy [16]. Clinical observation indicates that most cancer cells develop radioresistance during radiotherapy, sooner or later, especially in those treated with frequently small doses [16]. The present study mimicked this phenomenon with a cell culture model. By repeating 4 times of exposing NPC cells to small doses of irradiation (the 1×4 irradi), radioresistance was developed. We found that the defects of apoptosis occurred in NPC cells after irradiation. To induce cancer cell apoptosis is one of the major mechanisms of radiotherapy [24]. Since many therapies of cancer are to induce cancer cell apoptosis, including radiotherapy and chemotherapy [25], the defects of apoptosis can be a major obstacle in the therapeutic efficacy of radiotherapy in NPC and other cancers.

The data show that livin can be detected in NPC cells; this is in line with others’ reports; such as Xiang et al found high livin levels in the NPC tissues and suggested that the livin levels mirrored the prognosis of NPC [26]. Liu et al also found that the livin expression was increased in the NPC tissues after radiotherapy [27]. Livin was originally found in melanoma. Researchers found that livin could facilitate the melanoma growth by interfering the apoptotic machinery in melanoma cells. The present study also found this feature of livin in NPC cells. We further found that irradiation increased the expression of livin in NPC cells, indicating that the livin expression can be adjusted by irradiation in NPC cells. Such an adjustment results in the defects of apoptosis in NPC cells. The results also provide mechanistic evidence for this phenomenon. Radiation induces livin expression in NPC cells; livin interferes with the Fas gene transcription, and thus, induces the defects of apoptosis in NPC cells; this feature results in radioresistance in NPC cells.

Although the development of radioresistance is a natural phenomenon in biological cells [16], it still can be influenced, or adjusted by many factors. Such as inhibition of AKT signal pathway can overcome radioresistance and increase the sensitiveness of cancer cells to radiotherapy [28]. The present data show that the defects of apoptosis are the major feature of radioresistance in NPC cells. To adjust the defects of apoptosis and restore the apoptotic machinery in cancer cells can facilitate eliminating cancer cells from the body. The present study revealed that livin played a key role in the development of radioresistance by suppressing the Fas expression and thus, induced the defects of apoptosis in NPC cells. Therefore, to regulate the expression or the activities of livin is expected to prevent or inhibit the development of radioresistance in target cells. The reasoning is supported by the present data. By depletion of the expression of livin with RNAi approach, or the presence of livin inhibitors [19], can efficiently prevent radioresistance development. Furthermore, the established radioresistance in NPC cells also can be overcome by regulating the activities of livin.

## Conclusions

The present data show that livin plays a critical role in radioresistance development in NPC cells. To inhibit livin can prevent the radioresistance development or overcome the established radioresistance. Therefore, to regulate the expression of livin or inhibit the activities of livin has clinical translational potential to promote radiotherapy efficacy for NPC or other cancers.

## Supporting information

S1 Graph (PDF)
