# Supplementary material for: Inhibition of livin overcomes radioresistance in nasopharyngeal carcinoma cells
Source: PLoS One. 2020 Mar 2;15(3):e0229272. doi: 10.1371/journal.pone.0229272 (PMC7051067; doi:10.1371/journal.pone.0229272)

Supplemental materials (Full-length gel graphs)

Fig. 1D

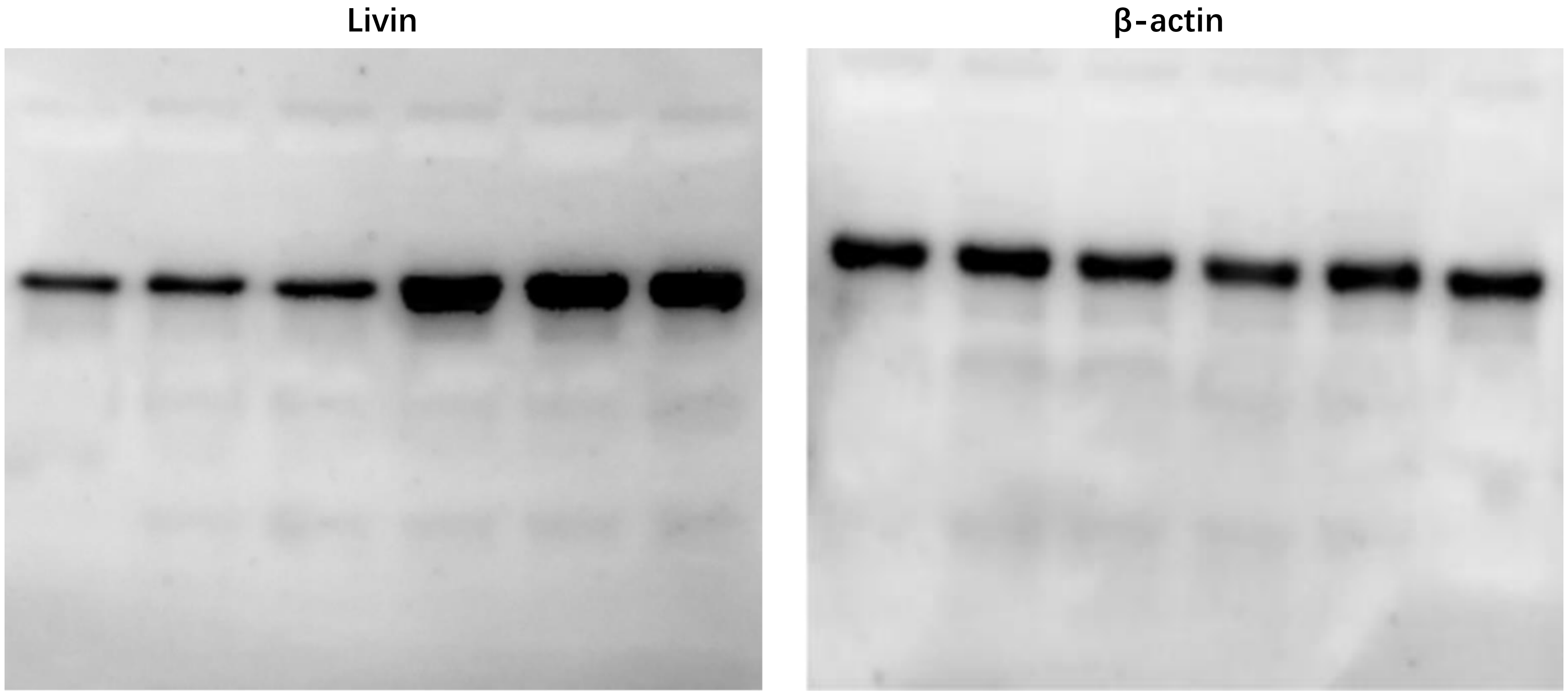

Fig. 3D

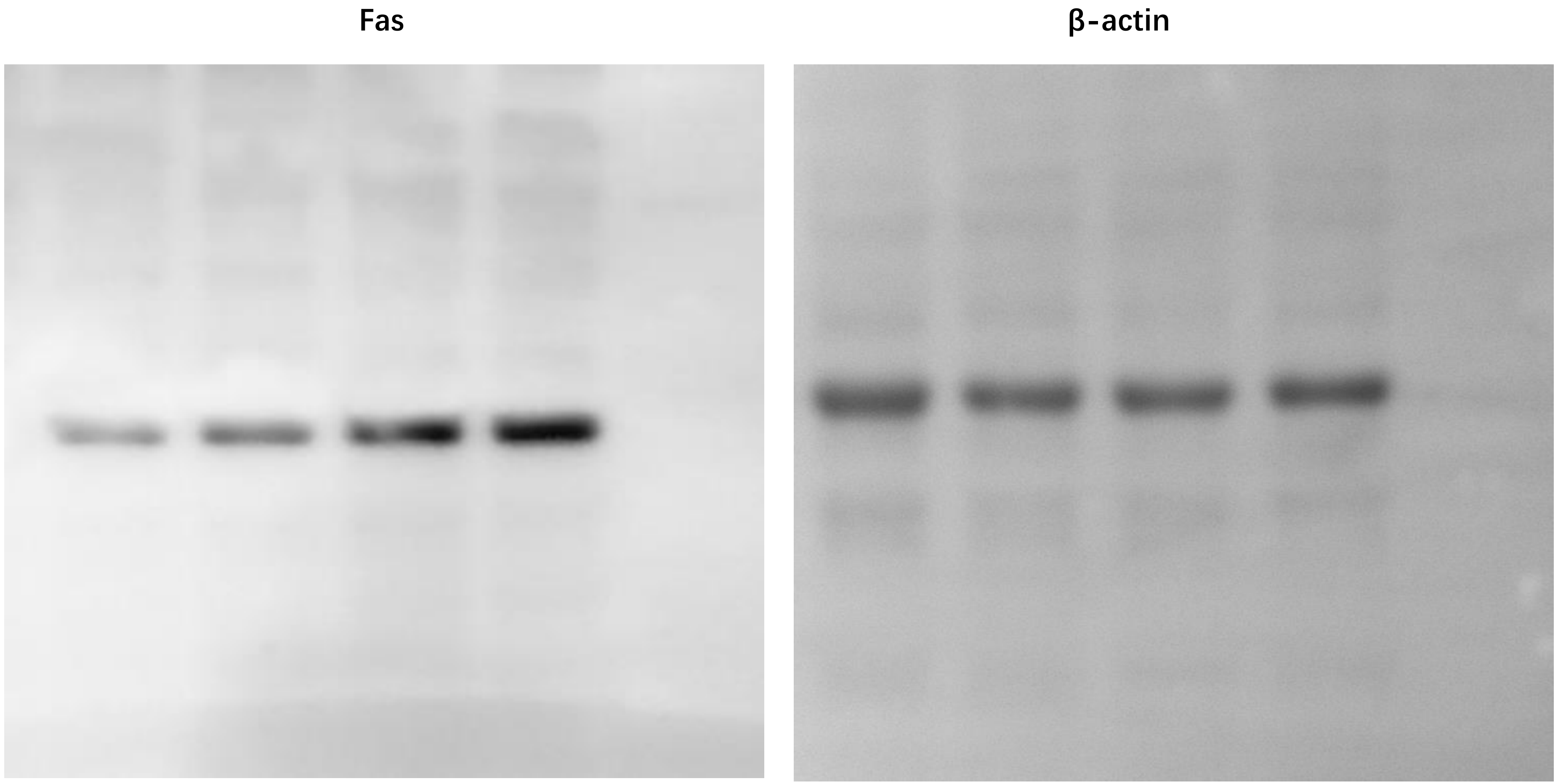

Fig. 4A

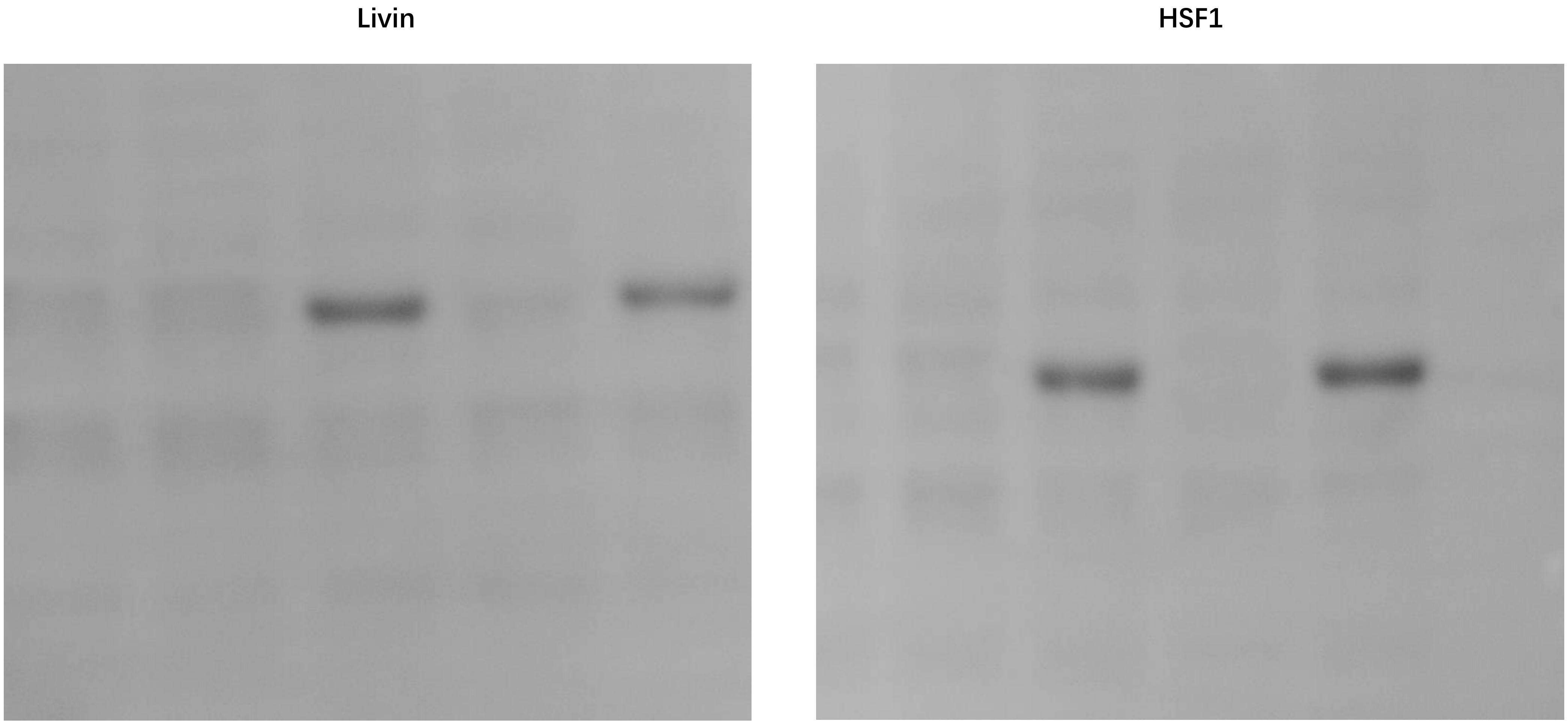

Fig. 4B

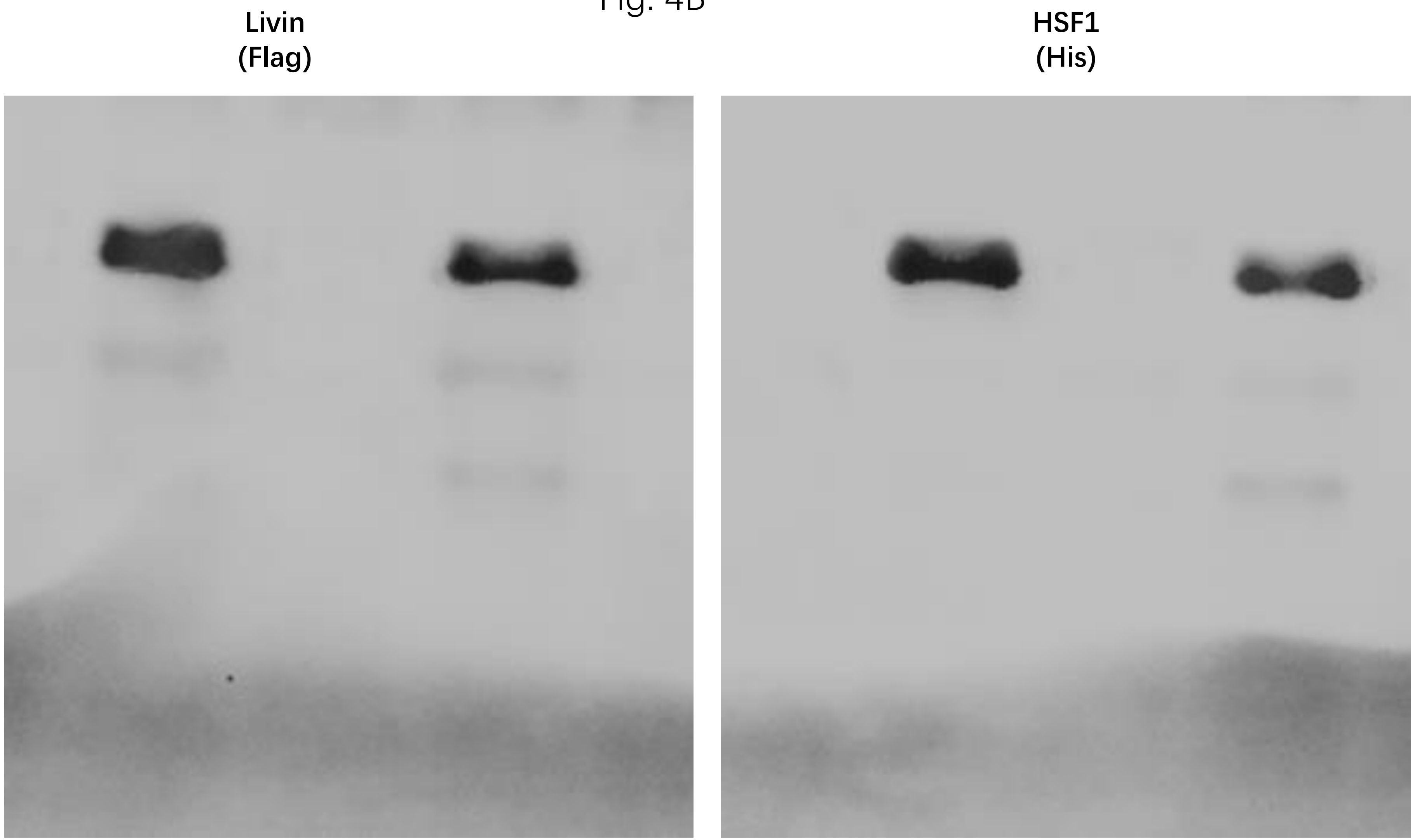

Fig. 4C

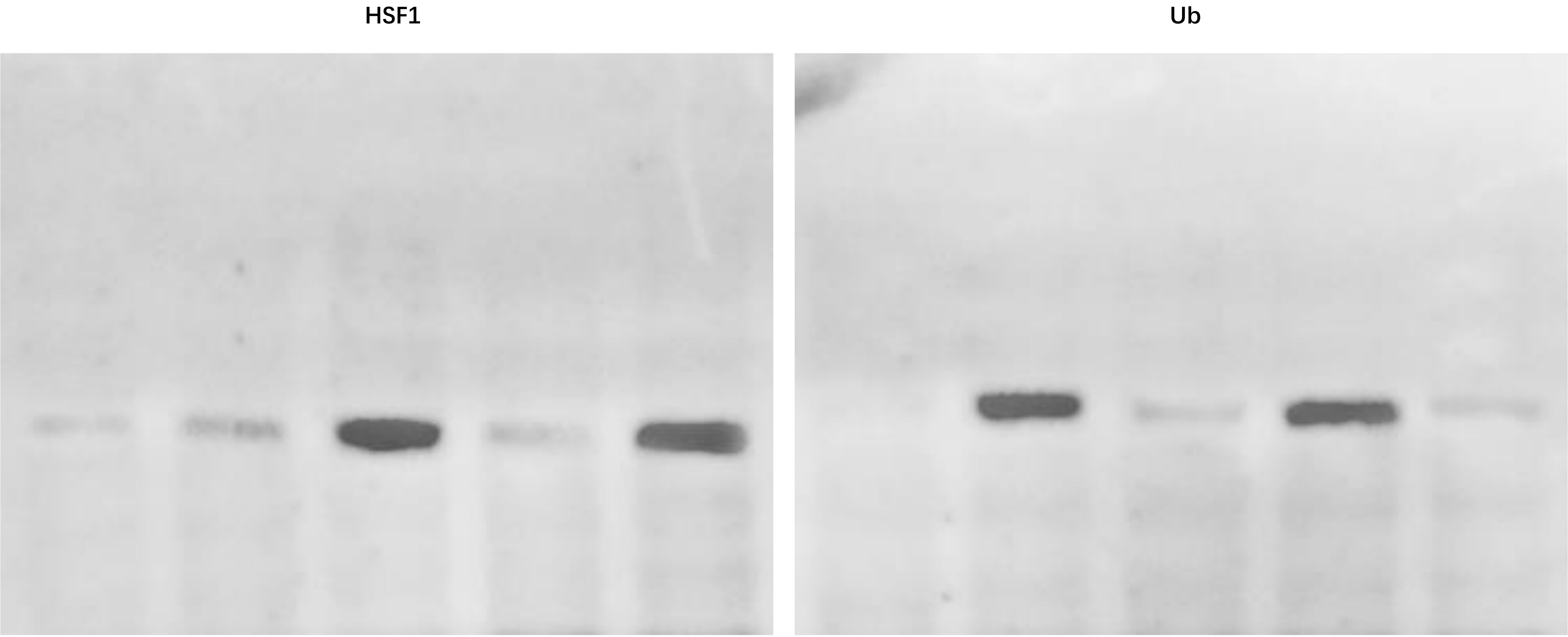

Fig. 4D

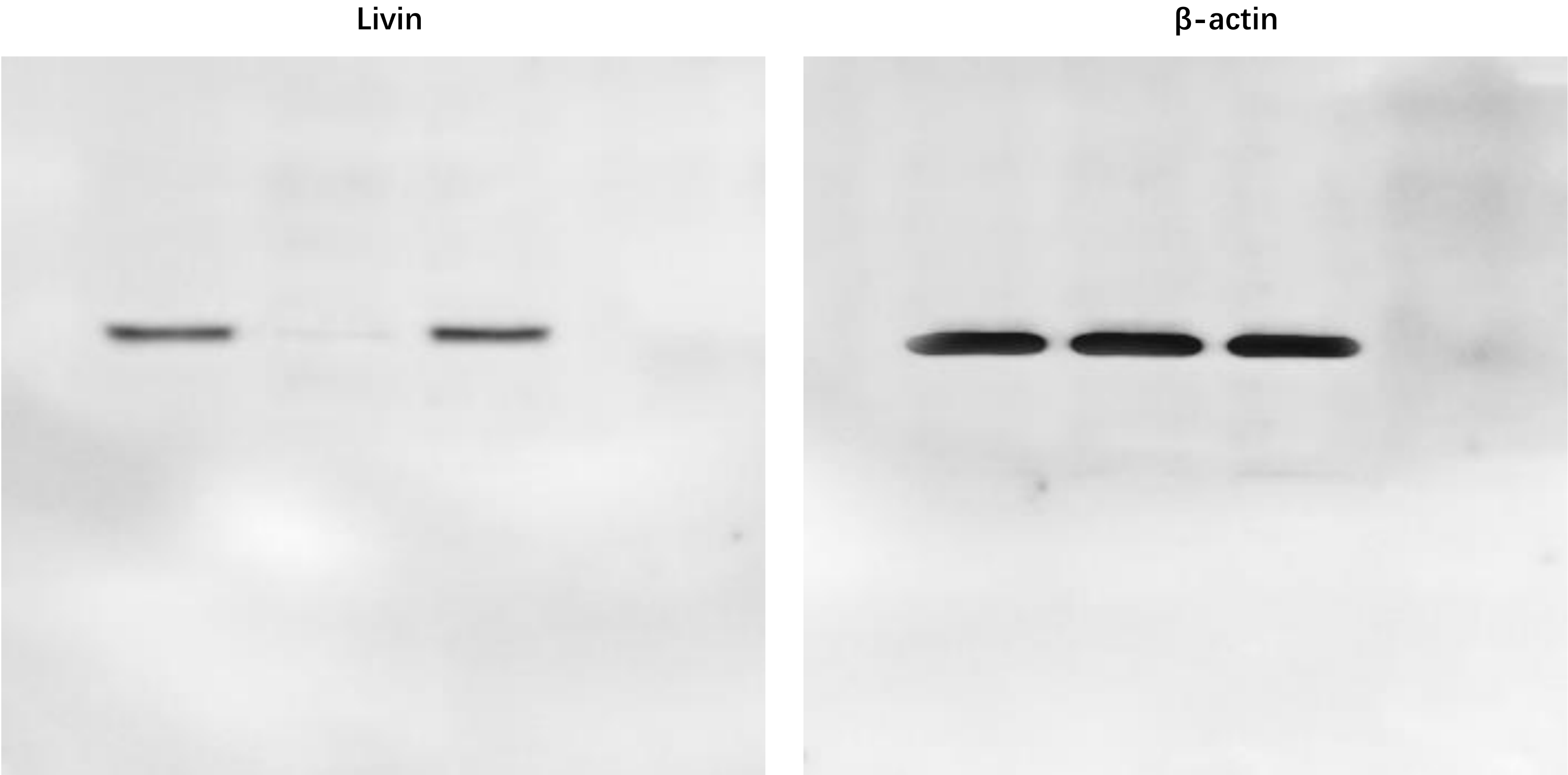

Supplement: S1 Graph — (PDF) [file pone.0229272.s001.pdf]
